# Study of secondary dentine deposition in central incisors as an age estimation method for adults

**DOI:** 10.1007/s12024-024-00777-9

**Published:** 2024-02-09

**Authors:** S. Marques-Moura, I. M. Caldas

**Affiliations:** 1 Department of Public Health and Forensic Sciences and Medical Education, Faculty of Medicine of the University of Porto, Al. Prof. Hernâni Monteiro, 4200-319 Porto, Portugal; 2 Faculty of Dental Medicine of the University of Porto, Rua Dr. Manuel Pereira da Silva, 4200-393 Porto, Portugal; 3 CFE – Center for Functional Ecology - Science for People and the Planet (CFE), University of Coimbra, Coimbra, Portugal; 4 1H-TOXRUN – One Health Research Unit in Toxicology, University Institute of Health Sciences, CESPU, CRL, 4585-116 Gandra, Portugal

**Keywords:** Forensic age estimation, Dental age estimation, Pulp/tooth area ratio, Secondary dentine, Upper incisors, Adults

## Abstract

This work aimed to assess the pulp/tooth area ratio’s utility in the upper central incisors using orthopantomograms. A convenience sample of 801 adult patient orthopantomograms was studied. Image J^®^ software was used to measure the pulp/tooth area ratio, and a regression model was developed. Our results conclude that the methodology assessing upper incisors’ pulp/tooth area ratio using orthopantomograms can lead to age overestimation and statistically significant differences between chronological and estimated age. For those over 50, no correlation between pulp/tooth area ratio and chronological age was found, suggesting that this may be the upper limit of this technique in this population. This methodology may not be suitable for age estimation, particularly in older adults.

## Introduction 

Age is one of the four major biological profile characteristics used to establish individual identification, with a growing essential role in the forensics [1]. 

The challenge of age estimation is higher in adults than in children since dental and skeletal growth is settled, and there is an increase in complexity as degenerative processes appear in adulthood [2, 3]. The most used indicators of chronological age achievement rely on skeletal and dental evaluations, considering the influence of environmental factors [4–6], ethnic and sexual variability [7], and a secular trend [8–10]. For age estimation, the Forensic Anthropology Society of Europe preconizes a radiograph of clavicle-sternal fusion, a dental study with pulp chamber methods, and a physical examination, including hormonal dosage for women. There is broad consensus that tooth assessment, relying on the dental age-related phenomena, is more predictable than the other two [11]. While comparing the radiation doses of the radiographs advocated for forensic proposals with natural and civilizing radiation exposures, it is accepted that the health risk of damage is diminutive. Exposure must respect the legislation of each country, recognizing the disparities between countries concerning purposes other than medical reasons. It is seen as a social and individual benefit in the majority, while radiation is taken for legal procedures. The imaging procedure must be performed under informed consent, including the purpose and examination type. On edge, images could be acquired from archives.

Dental age prediction in adults can rely on several methods, namely Gustafson’s parameters [12], dentinal translucency [13–22], and cementum annulations [23, 24]. Recent developments in biochemistry have allowed exact age estimation [5, 6]. However, these techniques require extraction of teeth and, usually, tooth sectioning/processing, which may not be feasible in living adults or in certain jurisdictions that prohibit tissue collection from human remains.

In 1925, Bodecker was the first author to recognize a correlation of dentine apposition with chronological age [25]. Secondary dentine apposition and pulp chamber narrowing since adulthood are well-recognized age indicators [11, 26]. After tooth full eruption, the apical closure is essential to begin secondary dentine secretion [26]. In the meantime, the pulp area decreases [27]. In 2004, Cameriere et al. introduced the pulp/tooth area ratio (PTAR) technique, measuring whole pulp and tooth areas and applying concrete age estimation statistical analysis, considering linear regression models [28]. This method, measuring the upper right canines in orthopantomograms (OPGs), has obtained high levels of accuracy in age prediction and included the effect of population affinity and culture on statistical formulation [28, 29]. It led to a simple and objective age estimation metric method, recommended for adults and individuals nearly to adulthood without third molars [26, 30]. Yet, some authors have claimed that PTAR models must be population-specific [31–34]. Later, in 2013, Cameriere et al. developed a model using peri-apical digital X-rays of both upper lateral and central incisors. The total variance explained by the model developed was the following: (a) 51.3% in lower lateral incisors, (b) 56.5% in lower central incisors, (c) 80.3% in upper central incisors, and (d) 81.6% in upper lateral incisors. The developed models were not tested in independent samples [27].

Furthermore, the study was carried out in peri-apical X-rays from skulls, which may not reproduce entirely the actual context and allow only visualization of a few teeth. This can be troublesome if the tooth the investigator was planning to assess cannot be evaluated (because it has a root canal treatment, for example) [28] and might require further radiographs to be performed. Recently, doubts have arisen about the ethics of these procedures [35], and it is a good practice to perform as few radiographs as possible [36]. Thus, an obvious advantage is using an orthopantomogram, which allows for multiple age estimation techniques. The study aimed to contribute to age estimation using the pulp/tooth area ratio in incisors assessed in orthopantomograms.

## Materials and methods

This research studied 801 patients’ OPGs. An Ethical statement was issued by the Ethical Commission of the Health Sciences of FMDUP (14/2022).

The selected individuals were European with Portuguese nationality and place of birth in Portugal. The presence of systemic and dental disorders was adopted as exclusion criteria. The teeth elected were the upper central incisors due to their favorable anatomy and because they house little environmental changes over a lifetime. Regarding teeth selection, only sound teeth were considered. The dental exclusion criteria were as follows: the presence of fillings, endodontic treatments, wear, fractures, impaction, extrusion, artifacts, developmental abnormalities, periodontal disease, peri-apical lesions, root resorption, open apex, multi roots, multi canals, pulp calcification, orthodontic treatment, moderate and severe superimposition, and rotation.

The analysis and selection of OPGs have considered the quality of the image, including the resolution features and absence of magnificence, noise, or artifacts. OPGs were classified into a wide range of groups by age, from 18 to 78 years old. Four hundred sixty-six belonged to female and 335 to male patients. The mean (M) chronological age (CA) of the participants was 37.01 years old (standard deviation (SD) = 15.10 years old). The median (Mdn) was 34.0 years old (interquartile range (IQR) = 24.0 years old). The patient distribution by age group and sex can be observed below (Table 1).

**Table 1:** Patients’ age and sex distribution included in the study (*n*, %)

**Age**		**Sex**		**Total**
		**Female**	**Male**	
** < 21**	*n*	33	32	65
	Sex, %	7.1%	9.6%	8.1%
	Total, %	4.1%	4.0%	8.1%
**21–30**	*n*	170	107	277
	Sex %	36.5%	31.9%	34.6%
	Total %	21.2%	13.4%	34.6%
**31–40**	*n*	82	70	152
	Sex, %	17.6%	20.9%	19%
	Total, %	10.2%	8.7%	19%
**41–50**	*n*	87	62	149
	Sex, %	18.7%	18.5%	18.6%
	Total, %	10.9%	7.7%	18.6%
**51–60**	*n*	51	31	82
	Sex, %	10.9%	9.3%	10.2%
	Total, %	6.4%	3.9%	10.2%
**61–70**	*n*	32	21	53
	Sex, %	6.9%	6.3%	6.6%
	Total, %	4.0%	2.6%	6.6%
** > 70**	*n*	11	12	23
	Sex, %	2.4%	3.6%	2.9%
	Total, %	1.4%	1.5%	2.9%
**Total**	*n*	466	335	801
	Sex, %	100.0%	100.0%	100.0%
	Total, %	58.2%	41.8%	100.0%

The PTAR measurements of both upper central incisors were performed without prior knowledge of the individual CA.

**Fig. 1:**
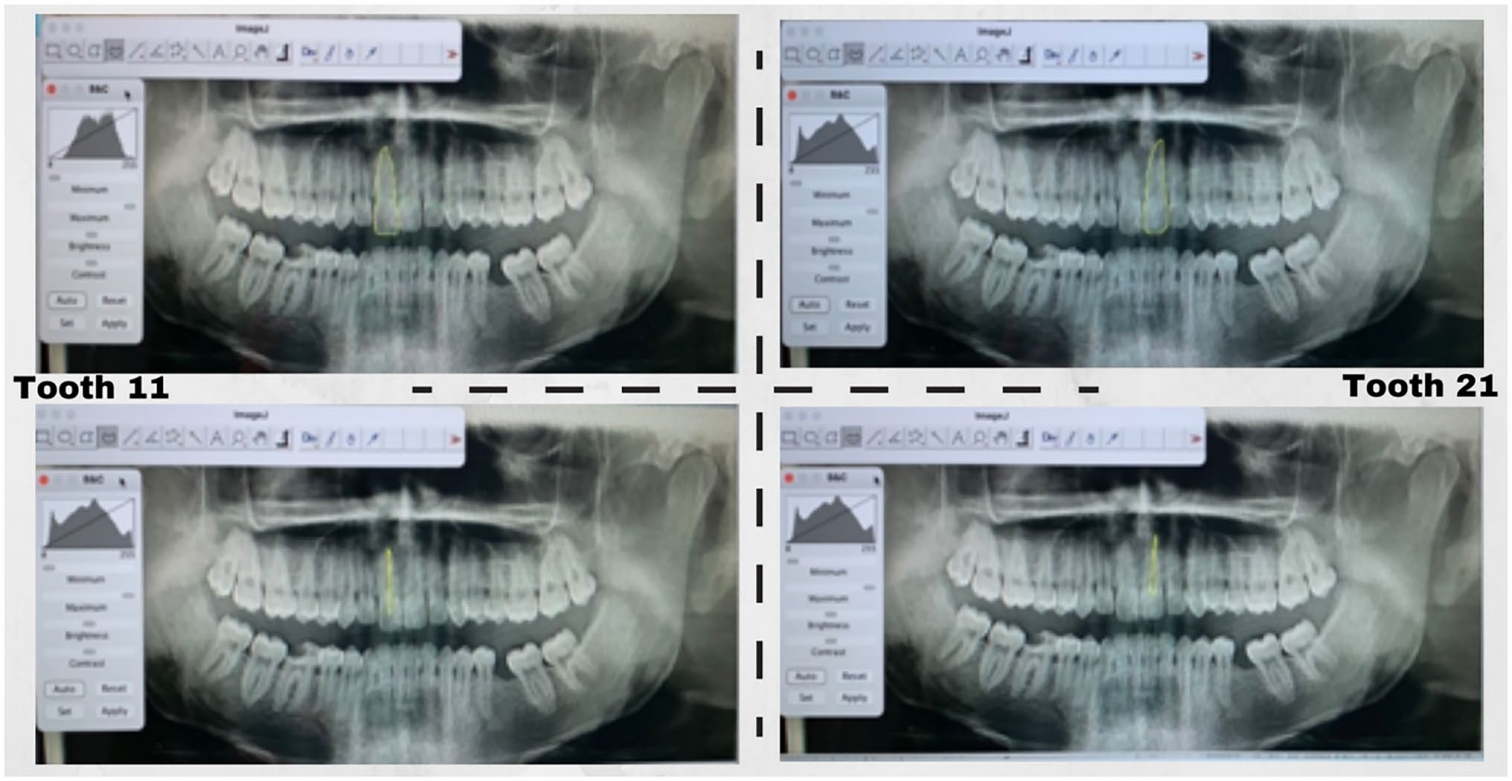
Manual tooth and pulp delineation, considering tooth laterality, using Image J^®^

The OPGs were obtained in JPEG format and numbered from one to 801. Image J^®^ software version 1.8.0 (open-source Java^®^-based image processing program developed by the National Institutes of Health and the Laboratory of Optical and Computational Instrumentation, LOCI, University of Wisconsin, USA) was used for semi-automatic area measurements. We have acquired area measurements using the “freehand selections” mode of Image J software to manually draw the pulp and tooth anatomical outlines (Fig. 1). About image optimization, the most adopted tool for the edition was the inversion, and the most used adjustment tools were contrast and brightness. Smoothing and sharpening processing tools were also very useful. Then, the pixel amount of each pulp and tooth area drawn was converted into areas automatically by the software, which performs the area calculation. Data were registered in Microsoft Excel^®^.

Statistical analysis was performed using the Statistical Package for Social Sciences program (SPSS), version 27.0. Reproducibility and repeatability were assessed using the Cronbach alpha coefficient by evaluating the agreement of the upper right central incisor (tooth 11) measurements in 30 randomly selected digital OPGs. The same OPG was examined three times for tooth and pulp measurements by the same observer (SMM), 2 days apart between each observation and by another (IMC). Normality was tested, resorting to the Kolmogorov-Smirnov test. As seen above, descriptive analysis has been performed for the continuous variable, resorting to M, SD, maximum (Max), minimum (Min) limits, Mdn, and IQR.

PTAR measurements were used for age estimation using Cameriere’s regression model [27]:

$$\text{Age}=78.55-3.86\cdot\text{g}-313.45\cdot\text{RA1sup}$$

where *g* is the sex [0, female and 1, male], and RA1sup stands for the PTAR of the upper central incisor.

The used model total variance (*R*^2^) is 0.803, and the standard estimate error (SE) is 7.03 years. As both upper central incisors were measured, we estimated age using 11(EA11) and 21(EA21).

The Pearson chi-square test was used to check possible associations between categorical variables. Spearman’s rho was used to analyze possible correlations. Estimated age (EA) using Cameriere’s equation was compared with CA. Resorting to linear regression, an age estimation model for the Portuguese population was developed. Then, the population sample was divided into six age groups (≤ 29, 30–39, 40–49, 50–59, 60–69, and 70–79 years old). Using a paired-sample *t*-test, EA with Cameriere’s method and the developed model were compared with CA in each group. The statistical significance level was set at 5%.

## Results

The Cronbach’s alpha values were 0.996 for inter-agreement and 0.991 for intra-agreement, both relatively high.

The Kolmogorov-Smirnov normality test showed a skewed distribution (*p* < 0.05). The M and Mdn of EA, using tooth 11, were 44.23 (SD = 7.27) and 44.45 years (IQR = 8.83), respectively. Using tooth 21, the M and Mdn of EA were 42.82 (SD = 7.73) and 43.14 years (IQR = 9.0), respectively (Table 2).

**Table 2:** Descriptive analysis of estimated age using PTAR method, considering both upper central incisors (in years)

**Teeth**	***n***	**Min.**	**Max.**	***M***	**SD**	**Mdn**	**IQR**
11	801	12.27	75.05	44.2321	7.27032	44.45	8.83
21	801	8.81	73.68	42.8160	7.73170	43.14	9.0

CA did not display a statistically significant association with the PTAR (*p* = 0.423). Yet, this link was present when we divided the sample by age groups (*p* < 0.001). As for the correlation between CA and EA, a moderate direct correlation was found using tooth 11 (*r* = 0.679) and slightly higher with tooth 21 (*r* = 0.706) (Table 3). This correlation was statistically significant (*p* < 0.001 for both).

**Table 3:** Spearman’s rho correlation test between CA and EA

		**Estimated age 11**	**Estimated age 21**
**Chronological age**	Correlation coefficient	.679^**^	.706^**^
	*p*	< .001	< .001
	*n*	800	801

^**^Statistical significance

Wilcoxon Signed Rank test was employed to compare CA and EA within groups and the total sample (Table 4). There were statistically significant differences in both cases (*p* < 0.001). The *Z*-score showed a slightly better relationship while using tooth 21.

**Table 4:** Wilcoxon signed rank test results comparing CA and EA

	**EA 11-CA**	**EA 21-CA**	**EA 11-CA groups**	**EA-CA groups**
***Z***	-15.283	− 13.378	− 15.305	− 14.130
***p*-value**	< .001	< .001	< .001	< .001

Using linear regression, a model for estimating age was developed. We observed sex and teeth laterality as possible confounding variables, and the variable sex was excluded, as it presented a low correlation value (*r* = 0.018) with CA. Conversely, a moderate negative correlation was found between CA and PTAR, using tooth 11 (*r* =  − 0.672) and tooth 21 (*r* =  − 0.687). The variables PTAR 11 and PTAR 21 were strongly correlated (*r* = 0.876). Yet, as the correlation did not pose an absolute contraindication and could increase the robustness of the prediction, they were both kept.

The assumptions for the model were checked, starting with the multicollinearity analysis. As mentioned, the independent variables presented a correlation with the dependent variable greater than 0.3 (*r* =  − 0.672 and *r* =  − 0.687). The tolerance (*t* = 0.233) and variance inflation factor (VIF = 4) were more significant than 0.1 and less than 10, respectively. Thus, the assumption of multicollinearity was not violated. The assumptions regarding outliers, normality, linearity, homoskedasticity, and independence of residuals were also verified. The model explained 49.3% (*R*^2^ = 0.493) of the variance of age, showing a statistical significance of the built model (*p* < 0.001). The PTAR 21 variable showed the most significant contribution to the developed equation. All variables presented statistical significance (*p* < 0.001) (Table 5).

**Table 5:** Test statistics for the proposed model

	**Unstandardized coefficients**		**Standardized coefficients**			**95% confidence interval for B**	
**Model**	*B*	Sth. error	*β*	*T*	Sig.	Lower bound	Upper bound
**(Constant)**	87.751	1.861		47.142	< 0.001	84.097	91.405
**PTAR 11**	− 204.133	35.107	− .304	− 5.815	< 0.001	− 273.046	− 135.221
**PTAR 21**	− 280.986	34.897	− .421	− 8.052	< 0.001	− 349.487	− 212.484

Paired-sample *t*-tests showed statistically significant differences between EA means, using Cameriere’s equation (EA11 and EA21) and using our model (EA3), with CA means in all age groups (Table 6).

**Table 6:** Mean age differences and correlations between CA and EA11, EA21, and EA3 for age groups

**Age groups**	Age	Mean	SD	Std. Error	*p*	Paired-sample correlations (with CA)	
						Correlation	*p*
** ≤ 29**	CA	22.53	2.61	0.15	-	-	-
	EA11	39.15	6.09	0.34	< 0.001	− .103	0.067
	EA21	38.91	6.44	0.36	< 0.001	− 0.044	0.430
	EA33	28.00	15.538	0.871	< 0.001	0.066	0.242
**30–39**	CA	34.28	3.21	0.26	-	-	-
	EA11	44.36	5.33	0.43	< 0.001	0.389	< 0.001
	EA21	43.97	4.72	0.38	< 0.001	0.442	< 0.001
	EA33	36.95	6.09	0.496	< 0.001	0.399	< 0.001
**40–49**	CA	43.93	2.89	0.23	-	-	-
	EA11	47.27	3.84	0.30	< 0.001	0.348	< 0.001
	EA21	47.39	3.84	0.30	< 0.001	0.329	< 0.001
	EA33	41.76	5.15	0.40	< 0.001	0.364	< 0.001
**50–59**	CA	53.75	2.61	0.28	-	-	-
	EA11	49.29	6.46	0.69	< 0.001	0.136	0.209
	EA21	49.40	5.81	0.62	< 0.001	0.104	0.339
	EA33	45.10	2.61	0.28	< 0.001	0.101	0.350
**60–69**	CA	64.91	3.08	0.46	-	-	-
	EA11	52.68	5.28	0.78	< 0.001	0.040	0.790
	EA21	52.07	5.60	0.83	< 0.001	0.080	0.597
	EA33	49.76	7.17	1.06	< 0.001	0.284	0.06
**70–79**	CA	71.85	0.337	47.142	-	-	-
	EA11	53.22	6.97	1.21	< 0.001	− 0.162	0.368
	EA21	53.72	6.69	1.17	< 0.001	− 0.056	0.756
	EA33	51.90	8.78	1.53	< 0.001	− 0.041	0.822

## Discussion

Age estimation in adults is particularly troublesome, as no developmental markers are available for these ages, and therefore, age estimation relies on senescence indicators. Yet, age-related changes and environmental factors often alter these indicators, making it virtually impossible to discriminate between older ages reasonably [11]. Our results point out that difficulty, as the statistically significative correlation between CA and EA (regardless of the model used), is lost in ages over 50 years, and all models underestimate age in all age groups. Other authors report similar difficulties in identical age groups, although using different teeth [37]. These results point out that PTAR, namely using central incisors, may not be suitable for age estimation over this age, and other methodologies should be used. Different results were obtained by Cameriere et al. [27], who successfully applied this method in individuals older than 70, suggesting that population differences may exist, and these should be considered when choosing the methodology for age estimation. Also, the selected tooth to apply the method might play an important role as most studies reporting accuracy in EA using PTAR refer to canines and lower premolars (Table 7) [28, 32, 37–46]. Another critical factor to consider is that Cameriere’s methodology was developed in apical radiographs, and forcing its use in orthopantomograms can lead to errors, as the incisors’ images are undoubtedly distorted. Yet, we have chosen to use orthopantomograms due to the possibility of selecting different methods using one radiograph alone.

**Table 7:** Review of the main results of articles related to PTAR method using 2D images (by year, population studied, and used teeth)

**Article**	**Year**	**Population**	**Teeth**	**Main results for age estimation**
Anastacio et al. [31]	2018	Portuguese	Second premolars	Not reliable
Azevedo et al. [32]	2014	Italian	Upper canines	Useful
Babshet et al. [33]	2010	Indian	Lower canine	Suboptimal results
Babshet et al. [34]	2011	Indian	Lower lateral incisor, canine and first premolar	Judicious use
Cameriere et al. [28]	2004	Italian	Upper right canine	Useful
Cameriere et al. [28]	2004	Italian	Second molar	Useful
Cameriere et al. [38]	2006	Italian	Upper canines	Useful
Cameriere et al. [29]	2007	Italian	Canines	Useful
Cameriere et al. [39]	2009	Portuguese	Canines	Useful
Cameriere and Ferrante [41]	2011	Not referred	Canines	Useful
Cameriere et al. [40]	2012	Spanish	Lower premolars	Useful; No sex differences
Cameriere et al. [27]	2013	Portugues e	Lateral and central incisors	Useful
De Luca et al. [43]	2011	Mexican	Canines	Useful
Jeevan et al. [37]	2011	Indian	Canines	Useful (up to 45 years old)
Juneja et al. [44]	2014	Indian	Upper canines	Useful
Lee et al. [45]	2017	Koreans	Lower second premolar	Useful; sex differences (better for females)
Zaher et al. [47]	2011	Egyptians	Upper incisors	Useful
Zelic et al. [46]	2020	Serbians	Lower premolars	Useful

Regarding estimating the age of the dead, an adaptation to the corpse’s state of preservation is required. Portable apical radiographs could make obtaining the best angle for the best image easier. However, other methods, such as the biochemical techniques [5, 6], Lamendin [14, 16–18], and Gustafson methods [12], are well established with good results.

In ages 30–49, a correlation between CA and EA was found, regardless of the model used. This is of little value, as it would be a requirement to know a person’s age before the age estimation process. Age was underestimated, and statistically significant differences between CA and EA means were determined. This was true for all age groups, suggesting that this methodology may be inadequate for age estimation in this population. Similar results were obtained by Jeevan et al. [37] found this methodology in canines useful up to age 45. On the other hand, Anastacio et al., who also studied a Portuguese population but applied the PTAR methodology on second premolars, found the methodology unreliable in all age groups [31].

As stated, there was an age overestimation until age 50, and from there on, an underestimation. This may happen because secondary dentine deposition is a finite process, and as time goes by and the pulp area diminishes, the quantity of secondary dentine deposited diminishes. Hence, the increase in tooth area is also lower. This means this method may also have an upper age use limit. This may differ in different populations and certainly with the used tooth, as explained in Table 7.

Many authors justify the selection of a specific tooth for PTAR based on technical issues, such as visibility in the X-ray, lower superimposition phenomenon, and the frequent presence of the tooth (and less damaged) [46], among others. Yet, other considerations should be made, namely the probable age frame and the population affinity. Regarding the existence of specific population formulas, other investigators also support this claim [33, 34], arguing that this approach considers the specific population correlation with secondary dentine deposition. We believe this to be true, but the choice of the tooth in which the methodology will be applied should also reflect this, as different populations for different age intervals may favor other teeth. Cameriere et al. [27] said that PTAR using incisors could be a helpful methodology in age estimation. However, if used in other teeth, the process offers better results, namely in canines [28, 39, 42] and premolars [40]. Yet, Zaher et al. [47] reported high levels of accuracy using upper incisors, supporting the idea that tooth choice matters.

The limitations of the present study include the low representativeness of older adults, which may have caused less accuracy for the older age group’s assessment. Additionally, regression equations will always overestimate the younger age group and underestimate the older age groups. In the future, the developed model should be tested in an independent sample.

## Conclusions

Our results conclude that the upper incisors’ pulp/tooth area ratio, using orthopantomograms, overestimated age, and statistically significant differences between chronological and estimated age, are present. For those over 50, no correlation between pulp/tooth area ratio and chronological age was found, suggesting that this may be the upper limit of this technique in this population.

## Key points

1. Age estimation in adults is a complex process
2. The pulp/tooth area ratio in incisors has been proposed as a valuable methodology for adult age estimation
3. In orthopantomograms, pulp/tooth area ratio analyses overestimate age
4. In orthopantomograms, pulp/tooth area ratio analysis does not work in people over 50

## Data Availability

Data are available upon request.

## Declarations

### Permissions

The Responsible for Access to Information of FMDUP issued permission to obtain orthopantomograms (04_2023) on the 30th of January 2023. The Data Protection Unit of the University of Porto gave a license to observe and analyze the orthopantomograms (A-15/2023) on the 12th of June 2023.

### Software

Image J^®^ Version 1.8.0.

## References

[1] Lo Re G, Zerbo S, Terranova MC, Pardo S, Midiri F, Argo A, et al. Role of imaging in the assessment of age estimation. Semin Ultrasound CT MR. 2019;40(1):51–5. 30686368 10.1053/j.sult.2018.10.010

[2] Hagen M, Schmidt S, Schulz R, Vieth V, Ottow C, Olze A, et al. Forensic age assessment of living adolescents and young adults at the Institute of Legal Medicine, Munster, from 2009 to 2018. Int J Legal Med. 2020;134(2):745–51. 31907616 10.1007/s00414-019-02239-2

[3] Cattaneo C. Ethics in forensic science: renewed commitments and a call for papers across the Forensic Science International Family. Forensic Sci Int. 2021;324:110831. 34058463 10.1016/j.forsciint.2021.110831

[4] Caldas IM, Cardoso HFV. Socioeconomic differences in permanent teeth mineralization of Portuguese girls and boys from Porto, Portugal. Anthropol Anz. 2021. 10.1127/anthranz/2021/1313 33595590

[5] Carneiro JL, Caldas IM, Afonso A, Cardoso HF. Examining the socioeconomic effects on third molar maturation in a Portuguese sample of children, adolescents and young adults. Int J Legal Med. 2017;131(1):235–42. 27761650 10.1007/s00414-016-1476-3

[6] Dinis AR, Teixeira A, Perez-Mongiovi D, Caldas IM. Fluctuating asymmetry in third molar agenesis as an aid to estimate socioeconomic status. Forensic Sci Med Pathol. 2023. 10.1007/s12024-023-00706-2 PMC11525242 37672167

[7] Cattaneo C, De Angelis D, Ruspa M, Gibelli D, Cameriere R, Grandi M. How old am I? Age estimation in living adults: a case report. J Forensic Odontostomatol. 2008;26(2):39–43. 22717788

[8] Heuze Y, Cardoso HF. Testing the quality of nonadult Bayesian dental age assessment methods to juvenile skeletal remains: the Lisbon collection children and secular trend effects. Am J Phys Anthropol. 2008;135(3):275–83. 18000887 10.1002/ajpa.20741

[9] Kaygisiz E, Uzuner FD, Yeniay A, Darendeliler N. Secular trend in the maturation of permanent teeth in a sample of Turkish children over the past 30 years. Forensic Sci Int. 2016;259:155–60. 26773225 10.1016/j.forsciint.2015.12.031

[10] Vucic S, de Vries E, Eilers PH, Willemsen SP, Kuijpers MA, Prahl-Andersen B, et al. Secular trend of dental development in Dutch children. Am J Phys Anthropol. 2014;155(1):91–8. 24912457 10.1002/ajpa.22556

[11] Cunha E, Baccino E, Martrille L, Ramsthaler F, Prieto J, Schuliar Y, et al. The problem of aging human remains and living individuals: a review. Forensic Sci Int. 2009;193(1–3):1–13. 19879075 10.1016/j.forsciint.2009.09.008

[12] Gustafson G. Age determination on teeth. J Am Dent Assoc. 1950;41(1):45–54. 15428197 10.14219/jada.archive.1950.0132

[13] Bang G, Ramm E. Determination of age in humans from root dentine transparency. Acta Odontol Scand. 1970;28:3–35. 5265990 10.3109/00016357009033130

[14] Ackermann A, Steyn M. A test of the Lamendin method of age estimation in South African canines. Forensic Sci Int. 2014;236(192):e1-6. 24445081 10.1016/j.forsciint.2013.12.023

[15] Baz A, Mantovani S, Ramos RP, Santos B, Grecco L, Goncalves G, et al. Age-at-death assessed with Lamendin’s original and population-specific models in a modern Brazilian osteological collection. J Forensic Odontostomatol. 2022;40(3):45–51. 36623297 PMC10266702

[16] De Angelis D, Mele E, Gibelli D, Merelli V, Spagnoli L, Cattaneo C. The applicability of the Lamendin method to skeletal remains buried for a 16-year period: a cautionary note. J Forensic Sci. 2015;60(Suppl 1):S177–81. 25413353 10.1111/1556-4029.12611

[17] Foti B, Adalian P, Signoli M, Ardagna Y, Dutour O, Leonetti G. Limits of the Lamendin method in age determination. Forensic Sci Int. 2001;122(2–3):101–6. 11672963 10.1016/s0379-0738(01)00472-8

[18] Gonzalez-Colmenares G, Botella-Lopez MC, Moreno-Rueda G, Fernandez-Cardenete JR. Age estimation by a dental method: a comparison of Lamendin’s and Prince & Ubelaker’s technique. J Forensic Sci. 2007;52(5):1156–60. 17645490 10.1111/j.1556-4029.2007.00508.x

[19] Megyesi MS, Ubelaker DH, Sauer NJ. Test of the Lamendin aging method on two historic skeletal samples. Am J Phys Anthropol. 2006;131(3):363–7. 16617435 10.1002/ajpa.20446

[20] Prince DA, Ubelaker DH. Application of Lamendin’s adult dental aging technique to a diverse skeletal sample. J Forensic Sci. 2002;47(1):107–16. 12064635

[21] Sasso A, Spalj S, Mady Maricic B, Sasso A, Cabov T, Legovic M. Secular trend in the development of permanent teeth in a population of Istria and the littoral region of Croatia. J Forensic Sci. 2013;58(3):673–7. 23067116 10.1111/j.1556-4029.2012.02301.x

[22] Zorba E, Goutas N, Spiliopoulou C, Moraitis K. An evaluation of dental methods by Lamendin and Prince and Ubelaker for estimation of adult age in a sample of modern Greeks. Homo. 2018;69(1–2):17–28. 29729834 10.1016/j.jchb.2018.03.006

[23] Kagerer P, Grupe G. Age-at-death diagnosis and determination of life-history parameters by incremental lines in human dental cementum as an identification aid. Forensic Sci Int. 2001;118(1):75–82. 11343858 10.1016/s0379-0738(00)00382-0

[24] Kvaal SI, Solheim T. Incremental lines in human dental cementum in relation to age. Eur J Oral Sci. 1995;103(4):225–30. 7552953 10.1111/j.1600-0722.1995.tb00164.x

[25] Kazmi S, Manica S, Revie G, Shepherd S, Hector M. Age estimation using canine pulp volumes in adults: a CBCT image analysis. Int J Legal Med. 2019;133(6):1967–76. 31471652 10.1007/s00414-019-02147-5 PMC6811669

[26] Marroquin TY, Karkhanis S, Kvaal SI, Vasudavan S, Kruger E, Tennant M. Age estimation in adults by dental imaging assessment systematic review. Forensic Sci Int. 2017;275:203–11. 28410514 10.1016/j.forsciint.2017.03.007

[27] Cameriere R, Cunha E, Wasterlain SN, De Luca S, Sassaroli E, Pagliara F, et al. Age estimation by pulp/tooth ratio in lateral and central incisors by peri-apical X-ray. J Forensic Leg Med. 2013;20(5):530–6. 23756528 10.1016/j.jflm.2013.02.012

[28] Cameriere R, Ferrante L, Cingolani M. Variations in pulp/tooth area ratio as an indicator of age: a preliminary study. J Forensic Sci. 2004;49(2):317–9. 15027553

[29] Cameriere R, Ferrante L, Belcastro MG, Bonfiglioli B, Rastelli E, Cingolani M. Age estimation by pulp/tooth ratio in canines by peri-apical X-rays. J Forensic Sci. 2007;52(1):166–70. 17209930 10.1111/j.1556-4029.2006.00336.x

[30] Dehghani M, Shadkam E, Ahrari F, Dehghani M. Age estimation by canines’ pulp/tooth ratio in an Iranian population using digital panoramic radiography. Forensic Sci Int. 2018;285:44–9. 29433010 10.1016/j.forsciint.2018.01.016

[31] Anastacio AC, Serras C, Vargas de Sousa Santos RF, Palmela Pereira C. Validation of Cameriere’s medical-legal age estimation method using seconds premolars in a Portuguese population. J Forensic Leg Med. 2018;60:30–4. 10.1016/j.jflm.2018.09.005 30241093

[32] Azevedo AC, Michel-Crosato E, Biazevic MG, Galic I, Merelli V, De Luca S, et al. Accuracy and reliability of pulp/tooth area ratio in upper canines by peri-apical X-rays. Leg Med (Tokyo). 2014;16(6):337–43. 25092574 10.1016/j.legalmed.2014.07.002

[33] Babshet M, Acharya AB, Naikmasur VG. Age estimation in Indians from pulp/tooth area ratio of mandibular canines. Forensic Sci Int. 2010;197(1–3):125 e1–4. 10.1016/j.forsciint.2009.12.065 20106616

[34] Babshet M, Acharya AB, Naikmasur VG. Age estimation from pulp/tooth area ratio (PTR) in an Indian sample: a preliminary comparison of three mandibular teeth used alone and in combination. J Forensic Leg Med. 2011;18(8):350–4. 22018166 10.1016/j.jflm.2011.07.003

[35] Thevissen PW, Kvaal SI, Willems G. Ethics in age estimation of unaccompanied minors. J Forensic Odontostomatol. 2012;30(Suppl 1):84–102. 23221269

[36] Perez-Mongiovi D, Teixeira A, Caldas IM. The radiographic visibility of the root pulp of the third lower molar as an age marker. Forensic Sci Med Pathol. 2015;11(3):339–44. 26105787 10.1007/s12024-015-9688-2

[37] Jeevan MB, Kale AD, Angadi PV, Hallikerimath S. Age estimation by pulp/tooth area ratio in canines: Cameriere’s method assessed in an Indian sample using radiovisiography. Forensic Sci Int. 2011;204(1–3):209 e1–5. 10.1016/j.forsciint.2010.08.017 20869824

[38] Cameriere R, Brogi G, Ferrante L, Mirtella D, Vultaggio C, Cingolani M, et al. Reliability in age determination by pulp/tooth ratio in upper canines in skeletal remains. J Forensic Sci. 2006;51(4):861–4. 16882230 10.1111/j.1556-4029.2006.00159.x

[39] Cameriere R, Cunha E, Sassaroli E, Nuzzolese E, Ferrante L. Age estimation by pulp/tooth area ratio in canines: study of a Portuguese sample to test Cameriere's method. Forensic Sci Int. 2009;193(1–3):128 e1–6. 10.1016/j.forsciint.2009.09.011 19854595

[40] Cameriere R, De Luca S, Aleman I, Ferrante L, Cingolani M. Age estimation by pulp/tooth ratio in lower premolars by orthopantomography. Forensic Sci Int. 2012;214(1–3):105–12. 21821373 10.1016/j.forsciint.2011.07.028

[41] Cameriere R, Ferrante L. Canine pulp ratios in estimating pensionable age in subjects with questionable documents of identification. Forensic Sci Int. 2011;206(1–3):132–5. 20724086 10.1016/j.forsciint.2010.07.025

[42] Cameriere R, Ferrante L, Belcastro MG, Bonfiglioli B, Rastelli E, Cingolani M. Age estimation by pulp/tooth ratio in canines by mesial and vestibular peri-apical X-rays. J Forensic Sci. 2007;52(5):1151–5. 17680998 10.1111/j.1556-4029.2007.00530.x

[43] De Luca S, Bautista J, Aleman I, Cameriere R. Age-at-death estimation by pulp/tooth area ratio in canines: study of a 20th-century Mexican sample of prisoners to test Cameriere’s method. J Forensic Sci. 2011;56(5):1302–9. 21496018 10.1111/j.1556-4029.2011.01784.x

[44] Juneja M, Devi YB, Rakesh N, Juneja S. Age estimation using pulp/tooth area ratio in maxillary canines-a digital image analysis. J Forensic Dent Sci. 2014;6(3):160–5. 25177137 10.4103/0975-1475.137047 PMC4142405

[45] Lee JH, Lee C, Battulga B, Na JY, Hwang JJ, Kim YH, et al. Morphological analysis of the lower second premolar for age estimation of Korean adults. Forensic Sci Int. 2017;281(186):e1–6. 29103902 10.1016/j.forsciint.2017.10.005

[46] Zelic K, Pavlovic S, Mijucic J, Djuric M, Djonic D. Applicability of pulp/tooth ratio method for age estimation. Forensic Sci Med Pathol. 2020;16(1):43–8. 32048137 10.1007/s12024-019-00200-8

[47] Zaher JF, Fawzy IA, Habib SR, Ali MM. Age estimation from pulp/tooth area ratio in maxillary incisors among Egyptians using dental radiographic images. J Forensic Leg Med. 2011;18(2):62–5. 21315299 10.1016/j.jflm.2010.12.004

